# Multi-compartmental modeling in Brian 2

**DOI:** 10.1186/1471-2202-16-S1-P19

**Published:** 2015-12-18

**Authors:** Marcel Stimberg, Dan F M Goodman, Romain Brette

**Affiliations:** 1 Sorbonne Universités, UPMC Univ. Paris 06, UMR_S 968, Institut de la Vision, Paris, F-75012, France; 2 INSERM, U968, Paris, F-75012, France; 3 CNRS, UMR_7210, Paris, F-75012, France; 4 Department of Electrical and Electronic Engineering, Imperial College London, London, UK

## 

Brian 2 is a fundamental rewrite of the popular Brian [1,2] simulator for spiking neural networks. It is written in the Python programming language and focuses on simplicity and extensibility: neuronal and synaptic models can be described using mathematical formulae and with the use of physical units [3].

Brian has been traditionally used to simulate networks of single-compartment neurons; complex morphologies, modeled as multi-compartment neurons, were not supported. We have recently added support for such models: a neuron's morphology can be either created from a file (in the SWC format [4]) or be created iteratively in the simulation script itself, using a convenient syntax. The flexibility of the Brian simulator applies to multi-compartment models in the same way as to single-compartment models: the model dynamics are governed by arbitrary user-provided differential equations, models can be flexibly parameterized over all the compartments and all variables describing the internal state of the model (voltages, currents, conductances, etc) are accessible to the user.

The simulation of multi-compartment models is more complex than that of non-electrically coupled single-compartment models: in addition to the numerical integration of the differential equations governing the change of the membrane potential in a single compartment, the currents flowing across neighboring compartments have to be taken into account. To this end, Brian employs a domain-decomposition method which corrects an initial solution valid for uncoupled compartments, taking the actual coupling into account. This method has the advantage that it is easy to parallelize, since most of computations are performed independently for each compartment. We demonstrate the advantage of this by parallelizing the computation over multiple processor cores using the OpenMP framework. For the future, we are planning to further accelerate the simulation speed by making use of the massively-parallel processing capabilities of graphical processing units.

With this addition, we have significantly enlarged the range of research questions that can be investigated using Brian.

Brian is made available under a free software license and all development takes place in public code repositories.
